# The role of gut microbiota mediated ferroptosis in PCOS and the therapeutic potential of Chinese herbal medicine

**DOI:** 10.3389/fmed.2026.1730795

**Published:** 2026-04-02

**Authors:** Yingchun Lv, Dongqi Li, Ning Ding, Hongying Kuang

**Affiliations:** 1 Graduate School, Heilongjiang University of Chinese Medicine, Harbin, Heilongjiang, China; 2 Second Department of Gynecology, The First Affiliated Hospital of Heilongjiang University of Chinese Medicine, Harbin, Heilongjiang, China

**Keywords:** ferroptosis, gut microbiota, polycystic ovary syndrome, traditional Chinese medicine compound formulas, traditional Chinese medicine monomers

## Abstract

Polycystic ovary syndrome (PCOS) is a complex reproductive endocrine metabolic disorder whose pathogenesis remains incompletely understood. In recent years, the role of ferroptosis–a novel form of iron-dependent programmed cell death–in the pathogenesis of PCOS has gradually drawn attention. This review proposes an innovative perspective: gut microbiota dysbiosis may be a potential upstream trigger of ferroptosis in PCOS ovarian granulosa cells. Microbiome dysbiosis disrupts iron homeostasis and reduces the production of antioxidant metabolites such as short-chain fatty acids (SCFAs) and bile acids (BAs), thereby exacerbating systemic and local ovarian oxidative stress. This induces ferroptosis, leading to impaired follicular development and insulin resistance. Traditional Chinese Medicine (TCM) demonstrates significant potential in regulating gut microbiota and inhibiting ferroptosis. Based on this, this study explores the role of the gut microbiota-ferroptosis axis in PCOS, focusing on the scientific rationale and application prospects of treating PCOS by intervening in this axis using TCM monomers and compounds such as berberine and quercetin. With its multi-target regulatory effects and favorable safety profile, TCM may offer benefits as an adjunct or alternative to conventional therapies. This research aims to provide theoretical references for developing novel therapeutic strategies.

## 1 Introduction

Polycystic ovary syndrome (PCOS) is one of the most common endocrine disorders in women of reproductive age, with a global prevalence of approximately 10%–13%. The clinical presentation typically includes hyperandrogenism (HA), ovulatory dysfunction, and polycystic ovarian morphology (1). The pathogenesis of PCOS involves dysregulation of the hypothalamic–pituitary–ovarian axis, insulin resistance (IR), and a chronic inflammatory state, and is influenced by genetic susceptibility, environmental factors, and metabolic disturbances. Nevertheless, the underlying mechanisms remain incompletely elucidated (2).

Ferroptosis is a recently identified form of programmed cell death driven primarily by iron-dependent lipid peroxidation and compromised cellular antioxidant defenses (3, 4). It has been implicated in the pathogenesis of a range of diseases, including cancer, (5) metabolic disorders, (6) and reproductive conditions (7). In the context of PCOS, emerging evidence indicates that ferroptosis activation aggravates mitochondrial dysfunction, oxidative stress, and inflammatory responses, which in turn contribute to follicular atresia, granulosa cell impairment, and diminished endometrial receptivity (8, 9).

The gut microbiota, meanwhile, exerts a profound influence on both PCOS development and ferroptosis through microbial metabolites and immune-inflammatory signaling pathways (10–12). This review therefore examines the potential involvement of gut microbiota-regulated ferroptosis in PCOS pathogenesis and systematically evaluates the unique advantages and therapeutic potential of Chinese herbal monomers and compound formulations in targeting this axis. Distinct from conventional approaches, Chinese medicine is characterized by its diverse mechanisms of action and reduced toxicity, supporting its application as either an adjunctive or primary therapeutic strategy. The findings are expected to offer new directions for understanding PCOS etiology and developing complementary treatment strategies.

## 2 Ferroptosis

### 2.1 The key role of iron metabolism and iron overload in triggering ferroptosis

Iron is an essential trace element that participates in fundamental physiological processes such as oxygen transport, energy metabolism, immune defense, and DNA synthesis. Dietary iron is absorbed primarily in the proximal small intestine, a process positively regulated by hypoxia-inducible factor-2α (HIF-2α) (13). Cellular iron homeostasis–governing its uptake, storage, and efflux–is critically dependent on the membrane protein ferroportin (FPN), which also plays a central role in ferroptosis (14). Systemic iron balance is predominantly controlled by hepcidin (Hepc), an antimicrobial peptide synthesized in the liver. Hepc reduces intestinal iron absorption by inhibiting HIF-2α and promotes cellular iron retention by inducing the degradation of FPN (15, 16). Consequently, the Hepc-FPN axis and the intestinal HIF-2α pathway are pivotal for maintaining systemic iron homeostasis. Dysregulation of these pathways can lead to iron dyshomeostasis, resulting in the accumulation of labile iron in tissues and, ultimately, iron overload–a condition increasingly recognized as a trigger for ferroptosis in various diseases, including PCOS.

### 2.2 Core characteristics of ferroptosis

Ferroptosis is an iron-dependent form of programmed cell death driven by lipid peroxidation. Its initiation depends on intracellular iron overload, which serves as the key biochemical basis and trigger, ultimately leading to extensive membrane damage. Morphologically and biochemically, ferroptosis is distinct from apoptosis, necrosis, and autophagy (17, 18). The execution of ferroptosis requires both the activation of specific lipid peroxidation pathways and a concurrent failure of the endogenous antioxidant defense system. Firstly, iron overload catalyzes the peroxidation of polyunsaturated fatty acids via the Fenton reaction, resulting in the excessive accumulation of lipid peroxides. Secondly, the synthesis of glutathione (GSH) is impaired, leading to the functional loss of its dependent antioxidant enzyme, glutathione peroxidase 4 (GPX4). This dual disruption prevents the effective clearance of lipid radicals (4, 19, 20). Consequently, uncontrolled, iron-dependent oxidative stress triggers the collapse of the cell membrane system and programmed cell death.

The cystine/glutamate antiporter system (System Xc^–^)/GSH/GPX4 axis represents a central pathway in the regulation of ferroptosis. System Xc^–^ is an antiporter composed of SLC7A11 and SLC3A2, which mediates the cellular uptake of extracellular cystine for GSH synthesis (21). GSH, the core antioxidant within this axis, serves as an essential cofactor for GPX4. It functions as an electron donor in redox reactions, cycling between its reduced (GSH) and oxidized (oxidized glutathione) states to mitigate oxidative stress. Among the GSH peroxidase family, GPX4 is uniquely critical in ferroptosis, acting as a key negative regulator by directly reducing lipid peroxides to suppress cell death (22). Beyond this core pathway, other molecular regulators–including the NAD(P)H/CoQ10/FSP1 axis, p53, Keap1-Nrf2, STAT3, and AMPK signaling pathways–also contribute to the modulation of ferroptosis (8, 23). Dysregulation of some of these pathways has been observed in PCOS, suggesting their potential involvement in the pathogenesis of the disease.

## 3 Ferroptosis in PCOS

### 3.1 Core pathophysiological features of PCOS and ferroptosis

Ovulatory dysfunction in PCOS often leads to oligomenorrhea or amenorrhea, which reduces iron loss through menstruation. Consequently, PCOS patients frequently exhibit elevated serum iron, ferritin, and transferrin saturation, indicative of a systemic iron-overloaded state (24, 25). In PCOS, IR and HA contribute to iron overload by downregulating Hepc expression, thereby diminishing its inhibition on FPN. This dysregulation promotes intestinal iron absorption and impairs iron release from macrophages (26). Furthermore, GPX4 expression is significantly downregulated in the tissues of PCOS patients. Inflammation-induced oxidative stress serves is a major factor contributing to GSH depletion and GPX4 inactivation (27). Concurrently, the aberrant expression of iron metabolism-related genes, along with intracellular iron accumulation–which is positively correlated with inflammatory responses–collectively promotes ferroptosis and subsequent tissue injury (27). Ultimately, the accumulated iron catalyzes the generation of reactive oxygen species (ROS) via the Fenton reaction, inducing lipid peroxidation and activating the ferroptosis pathway (28).

Iron deficiency, in turn, disrupts glucose homeostasis and promotes IR, thereby forming a vicious cycle with the metabolic disturbances characteristic of PCOS (29). Experimental evidence points to a potential link between ferroptosis and HA. For instance, animal studies have demonstrated that administration of the ferroptosis inducer RSL3 has been shown to elevate androgen levels in rats, a mechanism potentially mediated through the regulation of key steroidogenic enzymes such as CYP17A1, which plays a critical role in androgen biosynthesis and PCOS pathogenesis (30). The hallmark of ferroptosis–the accumulation of lipid peroxides and a compromised antioxidant system–further aggravates oxidative stress and chronic inflammation in PCOS. In summary, the core pathological features of PCOS, namely IR, HA, and a chronic inflammatory state, not only act as primary inducers of ferroptosis but are also amplified by the ferroptotic process itself. This interaction establishes a self-perpetuating vicious cycle that plays an important role in the initiation and progression of the syndrome.

### 3.2 Ovarian granulosa cell ferroptosis and impaired follicular development

Granulosa cells are the primary site of ovarian hormone synthesis and play a crucial role in regulating follicular development, oocyte maturation, and ovulation through hormone secretion. Studies have identified dysregulation of ferroptosis-related genes in granulosa cells from PCOS ovaries (31). Ferroptosis in PCOS granulosa cells is triggered through multiple mechanisms. Mechanistically, several pathways have been implicated. First, decreased expression of miR-128-3p in serum-derived exosomes from PCOS mouse models upregulates its target gene CSF1, which activates the p38/JNK signaling pathway and subsequently suppresses Nrf2-mediated transcriptional activity of SLC7A11. This cascade leads to ROS accumulation and lipid peroxidation, ultimately inducing ferroptosis (32). Second, HA enhances oxidative stress–particularly lipid peroxidation–and promotes intracellular iron accumulation in granulosa cells, thereby activating ferroptosis (33). while also triggering ferroptosis via NCOA4-mediated ferritinophagy and modulation of the GPX4 axis (30). Moreover, iron overload and excessive ROS production reduce mitochondrial membrane potential and cause mtDNA damage, further amplifying ROS generation. This forms a positive feedback loop with the aforementioned pathways, accelerating granulosa cell ferroptosis (34). Ultimately, ferroptosis in granulosa cells contributes to the disruption of follicular structure and function, which is associated with the clinical features of follicular developmental arrest and impaired oocyte quality in PCOS.

Ferroptosis contributes not only to the endocrine and metabolic disturbances in PCOS but also directly to its reproductive impairments. For example, HA and IR have been shown to trigger ferroptosis in the pregnant uterus and placental tissue (35). By disrupting systemic oxidative balance and compromising cellular integrity, ferroptosis adversely influences follicular development, ovulation, and hormonal regulation, thereby playing a critical role in the pathophysiology of PCOS.

## 4 Gut microbiota: a potential regulatory hub for ferroptosis in PCOS

### 4.1 Gut microbiota dysbiosis and host iron metabolism dysregulation

The gut microbiota comprises symbiotic microorganisms that colonize the human intestinal tract and interact with the ovaries through microbial metabolites, immune signaling, and neuroendocrine pathways. In PCOS, the pathogenesis of ferroptosis involves not only Hepc downregulation and subsequent increases in intestinal iron absorption but also the influence of gut microbiota and their metabolites. Microbial diversity is fundamental to maintaining gut ecosystem homeostasis and overall host health, with its abundance and stability directly shaping the adaptability of the intestinal microecosystem and host physiological function. Patients with PCOS display characteristic gut microbiota dysbiosis, though specific compositional alterations remain inconsistently reported across studies (36, 37). Numerous investigations have further revealed that PCOS is associated with reduced gut microbial diversity, which correlates closely with IR, sex hormone imbalances, and obesity (38, 39).

The gut microbiota and its metabolites play an important role in iron metabolism. Previous studies have demonstrated that the gut microbiota and its metabolites regulate iron metabolism by influencing iron absorption, transport, and storage. This occurs through altering the concentration of absorbable iron, modulating the intestinal absorption surface area, and affecting the expression of genes related to iron metabolism (40). For example, a dysbiotic microbiota can promote ferroptosis by suppressing SLC7A11 expression, which limits cysteine uptake and GSH synthesis, while concurrently enhancing acyl-CoA synthetase long-chain family member 4 (ACSL4)-driven lipid peroxidation (41). Additionally, microbiota-driven inhibition of the ornithine pathway represents another potential route for ferroptosis induction, which may contribute to the pathogenesis of PCOS. Conversely, host iron status reciprocally shapes the composition, abundance, and metabolic activity of the gut microbiota (42, 43). This bidirectional interaction between iron homeostasis and the gut ecosystem plays a significant role in the pathophysiology of PCOS.

### 4.2 Regulation of ferroptosis by gut microbiota metabolites

#### 4.2.1 Short-chain fatty acids

Short-chain fatty acids (SCFAs)–mainly acetate, propionate, and butyrate–have been shown to exert multiple beneficial effects in letrozole-induced PCOS rat models, such as improving lipid metabolism and mitigating oxidative stress and inflammation. They also modulate insulin secretion, resulting in lower fasting insulin levels and enhanced insulin sensitivity (44, 45). A study in sows further indicated that dietary SCFAs supplementation increases the number of secondary follicles while reducing follicular atresia and granulosa cell apoptosis (46). Recent studies have shown that butyric acid may regulate the expression of ovarian steroidogenic factors through the gut-brain-ovary axis, thereby improving follicular development and granulosa cell function, as well as ameliorating polycystic ovarian morphology (47). Therefore, SCFAs deficiency may represent a contributing factor to PCOS symptomatology, potentially through multiple interconnected mechanisms including exacerbating ovarian inflammation, impairing follicular maturation, aggravating IR, and promoting follicular atresia via direct suppression of glucose uptake in granulosa cells.

Studies have found that SCFAs may inhibit ferroptosis by activating the Keap1-Nrf2 antioxidant defense pathway and regulating iron metabolic homeostasis. Specifically, CFAs regulate Keap1 to activate Nrf2, upregulate GPX4 expression, enhance GSH-dependent lipid peroxide scavenging capacity, and preserve cell membrane integrity (48–50). Concurrently, SCFAs reduce iron absorption by inhibiting HIF-2α and upregulate ferritin expression to lower the labile iron pool, thus attenuating Fenton reaction-mediated lipid peroxidation (51). Furthermore, as energy substrates, SCFAs support mitochondrial respiration and enhance mitophagy to clear damaged mitochondria, reducing mtROS generation (49) –a mechanism particularly relevant in the context of PCOS-associated mitochondrial dysfunction.

Their anti-ferroptotic activity has also been experimentally validated: Huang et al. reported that butyrate protects granulosa cells by decreasing Fe^2 +^ and ROS levels while increasing GSH (52). Similarly, Chen et al. demonstrated that SCFAs exert anti-inflammatory and gut-barrier protective effects via the Nrf2/GPX4 pathway (53). However, gut dysbiosis in PCOS is associated with a decline in SCFAs-producing and BA-metabolizing bacteria, leading to a shift toward pro-inflammatory microbial communities and a reduction in anti-inflammatory taxa (54, 55). This imbalance attenuates endogenous protective mechanisms, ultimately disrupting ovarian iron homeostasis and amplifying lipid peroxidation damage–a cascade that may contribute to the follicular dysfunction characteristic of PCOS.

#### 4.2.2 Lipopolysaccharide

Lipopolysaccharide (LPS), a pro-inflammatory component derived from the outer membrane of Gram-negative bacteria, is found at elevated levels in the serum of PCOS patients and correlates positively with testosterone concentration, ovarian volume, antral follicle count, and hirsutism scores (56). Mechanistically, LPS not only triggers macrophage pyroptosis but also promotes apoptosis and disrupts estrogen synthesis in granulosa cells (57). With respect to iron metabolism, LPS suppresses hepatic Hepc expression through the TLR4/NF-κB pathway while upregulating the iron exporter FPN1 in duodenal enterocytes, thereby enhancing intestinal iron absorption (58). Gut-derived iron and inflammatory mediators can subsequently enter the systemic circulation and reach the ovarian microenvironment, where they exacerbate iron deposition and oxidative stress, ultimately impairing granulosa cell and oocyte function (59).

Lipopolysaccharide induces ferroptosis through multiple signaling pathways. Li et al. demonstrated that in human granulosa-like cells, LPS stimulation leads to alterations in ferroptosis-related protein expression, accompanied by the accumulation of ROS and Fe^2 +^. Further mechanistic studies revealed that LPS downregulates UFL1 expression, activates p53/SLC7A11 system, and modulates autophagy, thereby inducing ferroptosis (60). Additionally, Chen et al. found that in sheep ovarian granulosa cells, LPS treatment suppresses GPX4 expression, resulting in lipid peroxide accumulation and decreased mitochondrial membrane potential, while restoration of GPX4 expression reverses LPS-induced ferroptosis (61). These findings suggest that LPS may trigger ferroptosis in granulosa cells through a dual mechanism involving suppression of the antioxidant defense system (GPX4) and disruption of iron metabolic homeostasis (via SLC7A11-mediated cystine uptake and ferritin-mediated iron storage). Given that patients with PCOS often present with gut-derived endotoxemia, LPS-mediated granulosa cell ferroptosis may play a significant role in the pathogenesis of follicular developmental abnormalities in PCOS.

#### 4.2.3 Bile acids

Bile acids (BAs) metabolism disorders constitute a significant component of metabolic abnormalities in PCOS (62, 63). BAs exert regulatory effects on BAs homeostasis, glucose metabolism, and insulin sensitivity through activation of specific receptors, such as the farnesoid X receptor (FXR) and G protein-coupled receptors (64). FXR, a nuclear receptor activated by BAs, suppresses ferroptosis by upregulating the transcription of antioxidant genes such as GPX4 and FSP1; while TGR5, a membrane receptor, enhances mitochondrial function and reduces ROS generation via the cAMP-PKA signaling pathway, thereby indirectly lowering lipid peroxidation susceptibility (65). Furthermore, BAs contribute to the regulation of glucose and lipid metabolism through FXR and TGR5 activation (66). These findings underscore the importance of the gut microbiota–BAs–intestinal FXR axis in PCOS pathophysiology.

Notably, BAs -mediated activation of FXR has been reported to suppress lipid peroxidation and inhibit ferroptosis. A seminal study by Tschuck et al. demonstrated that activation of the nuclear receptor FXR by BAs significantly suppresses ferroptosis by upregulating multiple ferroptosis gatekeepers, including GPX4, FSP1, PPARα, SCD1 and ACSL3 (67). This coordinated regulation reduces lipid peroxidation and protects cells from ferroptotic cell death.

Furthermore, studies have confirmed the presence of physiologically relevant concentrations of BAs species in ovarian tissue, along with expression of FXR and BAs transporters (68). This provides the anatomical and biochemical basis for direct BAs signaling in ovarian granulosa cells. However, the functional consequences of BAs-FXR signaling specifically in ovarian granulosa cells remain to be fully elucidated. Given the established role of FXR activation in suppressing lipid peroxidation and the critical importance of granulosa cell integrity for follicular development, BAs may represent important endogenous regulators of ovarian function in PCOS.

#### 4.2.4 Lactic acid bacteria metabolites

Lactic acid bacteria produce various metabolites, including 1,3-diaminopropane (DAP), reuterin, and 5-methoxyindoleacetic acid (5-MIAA). Studies have shown that DAP and reuterin can inhibit the heterodimerization of HIF-2α with the aryl hydrocarbon receptor nuclear translocator, thereby downregulating the expression of intestinal iron absorption-related genes and subsequently limiting iron uptake (51). Studies have confirmed the expression of HIF-2α in granulosa cells, where it participates in regulating follicular angiogenesis and steroidogenesis (69). Therefore, DAP and reuterin may influence iron metabolism and ferroptosis sensitivity in granulosa cells by modulating local HIF-2α activity in the ovary; however, this hypothesis requires further experimental validation.

5-MIAA has been shown to activate Nrf2 (70). As a core transcription factor regulating the cellular anti-ferroptosis program, Nrf2 inhibits lipid peroxidation and the accumulation of labile iron by inducing the expression of its target genes, demonstrating significant potential in counteracting ferroptosis (71). Studies have confirmed that Nrf2 is expressed in ovarian granulosa cells and plays a critical role in oxidative stress response and iron homeostasis (72). These findings suggest that if 5-MIAA can be transported via the circulatory system to the ovarian microenvironment, it may exert similar Nrf2-mediated anti-ferroptotic effects in granulosa cells. Future studies are warranted to investigate whether DAP, reuterin, and 5-MIAA can reach the ovarian follicle and modulate ferroptosis pathways in granulosa cells, which may have important implications for understanding PCOS-associated follicular dysfunction. In PCOS patients, the reduced abundance of *Lactobacillus* leads to decreased circulating levels of these metabolites, which in turn attenuates the inhibition of intestinal iron absorption (63). This may represent one of the factors contributing to granulosa cell ferroptosis in PCOS.

#### 4.2.5 Oxidative stress and inflammatory response

An increased abundance of Gram-negative bacteria in the gut of PCOS patients elevates circulating LPS, which activates the LPS/TLR4 pathway and sustains systemic chronic low-grade inflammation (73). This inflammatory milieu upregulates hepatic Hepc expression, (74) leading to FPN degradation and subsequent iron retention in enterocytes and macrophages. The resulting expansion of the labile iron pool creates a permissive environment for ferroptosis.

During PCOS progression, persistent inflammation also induces mitochondrial dysfunction, uncouples the electron transport chain, and promotes substantial ROS leakage (75). Concurrently, decreased levels of antioxidant antimicrobial metabolites in PCOS further compromise cellular reducing capacity and intensify redox imbalance. ROS directly attack polyunsaturated fatty acids in cell membranes, initiating lipid peroxidation cascades (76). When lipid peroxide generation exceeds the clearance capacity of GPX4, irreversible damage to membrane integrity occurs, culminating in ferroptosis. In the PCOS ovarian microenvironment, key functional cells such as granulosa cells are particularly vulnerable to this pathway, which directly impairs follicular development and maturation. These findings suggest that the gut microbiota and their metabolites may participate in the pathogenesis of polycystic ovary syndrome by regulating ferroptosis; however, the direct causal relationship requires further validation.

#### 4.2.6 Interplay and hierarchical roles of microbial metabolites

**FIGURE 1:**
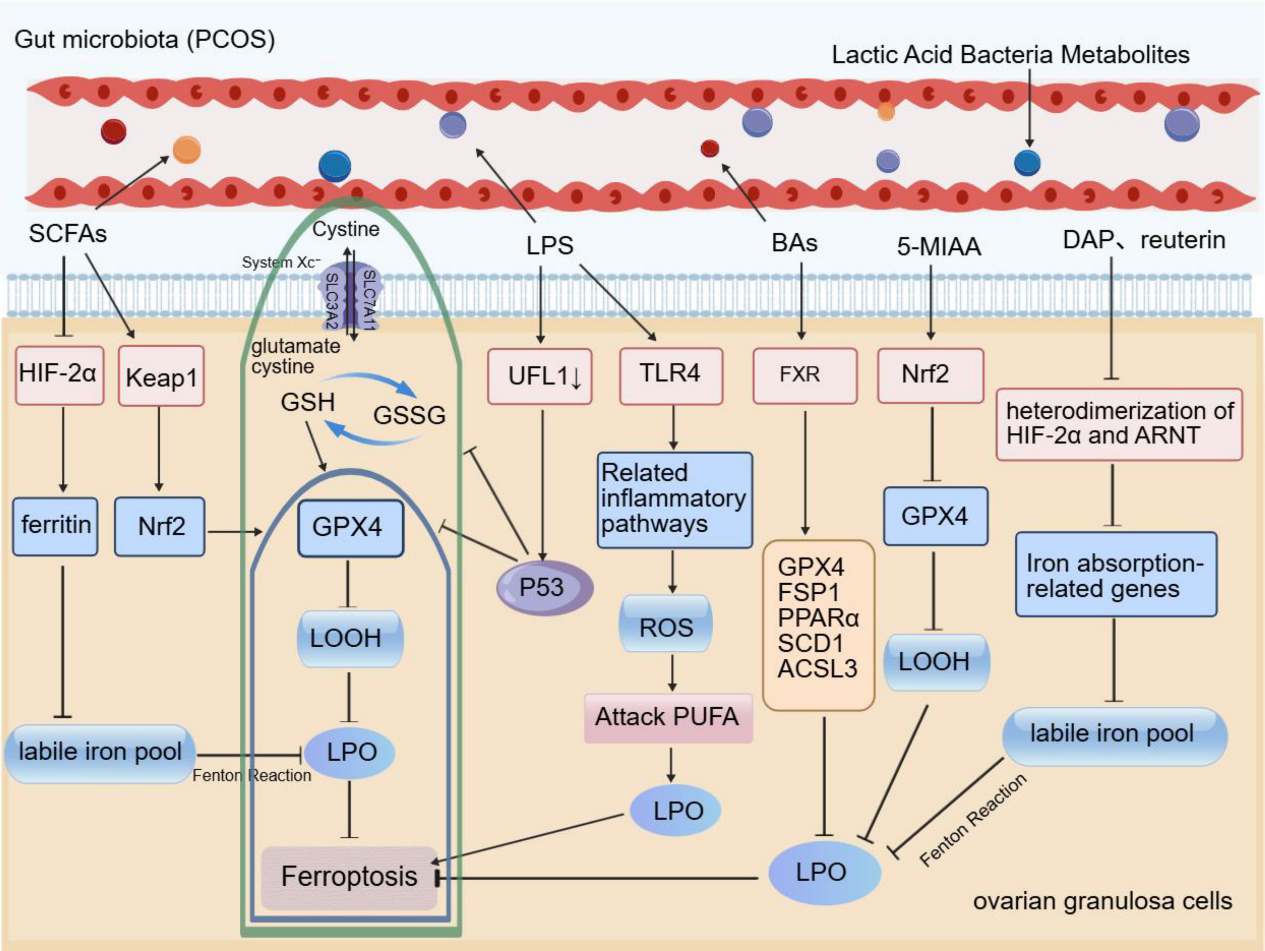
Schematic diagram of gut microbiota metabolites regulating ferroptosis in ovarian granulosa cells in PCOS. The black T-shaped arrows indicate inhibition or downregulation, while the black arrows indicate activation or promotion. The yellow background highlights the ovarian granulosa cells, the green border encloses the classical System Xc/GSH/GPX4 ferroptosis signaling pathway, and the blue border represents partially related regulatory processes of this pathway. GSSH, Oxidized glutathione; LOOH, lipid peroxide; LPO, lipid peroxidation; PUFA, polyunsaturated fatty acids.

Gut microbiota dysbiosis in PCOS patients leads to a reduction in bacteria that produce SCFAs and metabolize BAs, resulting in decreased levels of protective metabolites such as SCFAs and BAs. Concurrently, increased abundance of Gram-negative bacteria elevates levels of pro-inflammatory LPS. Although these metabolites change in opposite directions, their combined effect converges on promoting ferroptosis. Notably, systematic research and direct evidence regarding the primary and secondary roles of different metabolites in PCOS-associated ferroptosis remain lacking in the current literature. Based on available evidence, SCFAs exert protective effects by activating the Nrf2/GPX4 pathway to inhibit lipid peroxidation, while LPS acts through the dual mechanisms of suppressing the antioxidant defense system (GPX4) and disrupting iron metabolic homeostasis. Together, these two may serve as primary pro-ferroptotic drivers. Although BAs can regulate ferroptosis-related molecules via FXR activation and Lactobacillus metabolites possess Nrf2 activation potential, direct experimental evidence for their effects in PCOS ovarian granulosa cells is still lacking; therefore, they currently may function as secondary modulators. This proposed hierarchical framework requires validation through systematic metabolomic and functional studies to establish the relative contributions of different metabolites, thereby informing targeted therapeutic strategies (Figure 1).

## 5 The potential of TCM targeting the gut microbiota-ferroptosis axis for treating PCOS

Traditional Chinese Medicine offers unique advantages in the treatment of PCOS through its holistic, multi-component, and multi-target regulatory actions, particularly in modulating the gut microbiota. Based on syndrome differentiation and treatment principles, TCM employs individualized herbal formulations to achieve precision therapy. This integrative strategy, combining systemic regulation with personalized medicine, provides a safer and more effective treatment option for patients with PCOS. Accumulating evidence indicates that Chinese herbal compounds, active constituents, and formulated prescriptions can ameliorate ferroptosis in PCOS by remodeling the gut microbiota and modulating ferroptosis-associated pathways (Table 1).

**TABLE 1:** The potential of TCM targeting the gut microbiota-ferroptosis axis for treating PCOS.

Category	TCM compound/monomer	Botanical source or composition	Mechanism of action
Flavonoids	Quercetin	Numerous Chinese medicinal plants	Alleviates inflammation and intestinal injury, regulating ferritin autophagy and intracellular iron efflux
	Baicalin	*Scutellaria baicalensis* roots	Upregulating GPX4 expression and downregulating ACSL4 levels mitigate lipid peroxidation in ovarian granulosa cells
	Atractylodin	*Atractylodes macrocephala*	Inhibits granulosa cell ferroptosis via PDK4/JAK-STAT3 axis
	Plumbagin	*Plumbago zeylanica* roots	Suppressing the expression of YTHDF1
Alkaloids	Berberine	Numerous Chinese medicinal plants	Enriches butyrate-producing bacteria, modulating hepc activity, Regulation of the circ_0097636/miR-186-5p/SIRT3 pathway
	Nuciferine	Lotus leaves	Inhibits ferroptosis via the SOX2/SLC7A11/GPX4 axis
	Leonurine	Leonuri Herb	Modulates gut microbiota, increases butyrate, inhibits granulosa cell ferroptosis
Polysaccharide	*Astragalus* polysaccharide	The stems or dried roots of *Astragalus*	Protects intestinal barrier, promotes beneficial microbiota and SCFAs production
	Moutan Cortex	The dried root bark of *Paeonia lactiflora*	Enhances gut barrier and SCFAs, regulating iron absorption
Saponins	Salidroside	*Rhodiola rosea*	Improves gut microbiota, limits iron overload and downregulates iron metabolism genes
Polyphenols	Curcumin	Curcumin	Modulates gut microbiota-SCFAs barrier axis and inhibits LPS-induced inflammation
Others	Salicin B puerarin and astragaloside A		Bioinformatics-predicted association with ferroptosis targets
TCM Compound	Wenshen Tiaojing Decoction		Regulation of oxidative stress and ferroptosis
	Huanglian Jiedu Tang		Remodeling gut microbiota and modulating ferroptosis pathways
	Bushen Huatan Formula		Ameliorates gut dysbiosis, activates SCFAs-PPARγ pathway, and enhances gut barrier
	Shoutai Pill		Reduces lipid peroxidation and iron accumulation

### 5.1 TCM monomer

#### 5.1.1 Flavonoids

Quercetin, a naturally occurring flavonoid, demonstrates a range of pharmacological properties, such as antioxidant, anti-inflammatory, antidiabetic, and anticancer activities (77). It has been shown to inhibit LPS-induced expression of inflammatory cytokines and alleviate intestinal barrier injury by suppressing specific inflammasome activation (78). Furthermore, quercetin counteracts ferroptosis under inflammatory conditions by modulating the IL-6/STAT3 pathway to regulate ferritinophagy and promote cellular iron export (79). Together, these mechanisms help ameliorate PCOS-related symptoms and limit intestinal iron absorption.

Baicalin, a flavonoid derived from the roots of *Scutellaria baicalensis*, possesses a broad spectrum of pharmacological effects, including anti-inflammatory, antitumor, antibacterial, lipid-lowering, and antioxidant activities (80). In the context of ferroptosis, ACSL4 and GPX4 serve as key promotor and suppressor proteins, respectively. Studies indicate that baicalin attenuates lipid peroxidation in ovarian granulosa cells by upregulating GPX4 and downregulating ACSL4 expression, thereby inhibiting ferroptosis (81).

Atractylodin, the principal bioactive compound in *Atractylodes macrocephala*, possesses a range of pharmacological properties, including anti-inflammatory, antitumor, cardioprotective, and antioxidant activities (82). Zhou et al. (23) reported that atractylodin suppresses ferroptosis in ovarian granulosa cells via the pyruvate dehydrogenase kinase 4-mediated JAK-STAT3 signaling pathway, suggesting a potential mechanism through which it may exert therapeutic effects in PCOS.

Plumbagin, a natural naphthoquinone derived from *Plumbago zeylanica* roots, exhibits multiple pharmacological effects such as antioxidant, antidiabetic, antifungal, and anticancer activities (83). Recent studies demonstrate that plumbagin alleviates dihydrotestosterone-induced mitochondrial dysfunction and ferroptosis in granulosa cells by inhibiting YTHDF1 expression, thereby improving cell survival and function. Mechanistically, plumbagin interferes with the YTHDF1-mediated m^6^A methylation pathway that regulates SLC7A5 translation (84).

#### 5.1.2 Alkaloids

Berberine, an isoquinoline alkaloid present in numerous Chinese medicinal plants, exhibits diverse pharmacological effects including antibacterial, antidiabetic, and anticancer activities (85). Its potential in modulating ferroptosis in PCOS has attracted increasing research interest. Berberine not only enriches butyrate-producing bacteria and elevates butyrate levels, thereby improving gut microbiota composition and function, (86) but also attenuates inflammatory responses and endoplasmic reticulum stress induced by HIV protease inhibitors. It downregulates inflammatory mediators such as IL-6, indirectly modulating hepc activity and restoring iron homeostasis (87, 88). Additionally, berberine ameliorates granulosa cell injury and ferroptosis through the circ_0097636/miR-186-5p/SIRT3 pathway (89).

Nuciferine, an alkaloid derived from lotus leaves, is used clinically to manage hyperlipidemia and as an adjunct for weight control, owing to its lipid-regulating, insulin-secreting, vasodilatory, antihypertensive, antitumor, and immunomodulatory properties (90). Recent studies have further identified its protective role in PCOS. Yang et al. (91) demonstrated that nuciferine inhibits ferroptosis via the SOX2-mediated SLC7A11/GPX4 signaling axis, thereby protecting ovarian granulosa cells from androgen-induced damage.

Leonuri Herb, a plant of the Lamiaceae family, is medicinally derived from its dried aerial parts. Its primary bioactive constituents include alkaloids and diterpenoids. Studies indicate that the alkaloids in Leonuri Herb possess diverse pharmacological properties, such as diuretic, antiplatelet, anti-inflammatory, and antioxidant activities (92). Notably, leonurine, a major alkaloid component, modulates the gut microbiota by enhancing the abundance of beneficial bacteria and raising butyrate levels. These changes subsequently inhibit ferroptosis in ovarian granulosa cells, ultimately improving IR and ovarian function, which contributes to its therapeutic efficacy in PCOS (52).

#### 5.1.3 Polysaccharide

*Astragalus* polysaccharide (APS) is a water-soluble heteropolysaccharide extracted from the stems or dried roots of *Astragalus* membranaceus and represents the most abundant and biologically important component of this herb. Research indicates that APS helps protect the intestinal barrier, promotes the growth of beneficial gut microbiota, inhibits pathogenic bacterial colonization, and increases levels of SCFAs; It also exhibits anti-inflammatory activity by suppressing the NF-κB signaling pathway and downregulating pro-inflammatory cytokines (93). In PCOS treatment, APS has been shown to reduce blood glucose, improve insulin sensitivity, and inhibit pancreatic β-cell apoptosis (94).

Moutan Cortex, the dried root bark of *Paeonia lactiflora*, exhibits anti-inflammatory, immunomodulatory, and cardiovascular protective properties (95). Studies show that its active constituent paeoniflorin enhances intestinal barrier integrity and attenuates inflammatory responses by elevating SCFAs levels and reducing branched-chain amino acids (96). The resulting increase in SCFAs contributes to the regulation of intestinal iron absorption, thereby lowering the risk of systemic iron overload.

#### 5.1.4 Saponins

Salidroside (SAL), a major bioactive compound extracted from *Rhodiola rosea*, possesses a broad spectrum of pharmacological activities. These include protective effects on the respiratory, reproductive, and cardiovascular systems, along with anti-hypoxic, anti-aging, anticancer, anti-inflammatory, and antioxidant properties (97). In terms of gut microbiota modulation, SAL increases the abundance of probiotics such as Bacteroidetes and Firmicutes while reducing *Lactobacillus* levels, thereby improving microbial composition. Notably, *Lactobacillus* has been reported to promote intestinal iron absorption (98). In the context of iron metabolism, Shi et al. (99) demonstrated that SAL limits iron accumulation in diabetic mice and downregulates iron metabolism-related genes SLC7A11 and LC3II, effectively mitigating iron overload.

#### 5.1.5 Polyphenols

Polyphenols are a class of plant-derived compounds with potent antioxidant activity. Scarano et al. (100) reported that the presence of catechol and galloyl groups in their molecular structures enables plant polyphenols to exhibit strong iron-binding capacity. This iron-chelating property contributes to inflammatory and immune regulation, promotes gut microbiota remodeling, and alleviates iron overload along with associated oxidative cellular stress. For instance, curcumin–a natural polyphenol derived from turmeric rhizomes–modulates the gut microbiota by enhancing SCFAs production, inhibiting LPS-induced release of inflammatory factors, and improving intestinal barrier function (101). Further investigations demonstrate that, when combined with fecal microbiota transplantation, curcumin effectively restores microbial balance in PCOS rats, characterized by increased abundances of *Lactobacillus*, *Bifidobacterium*, and butyrate-producing bacteria (102).

#### 5.1.6 Others

A bioinformatics analysis based on the Coremine Medica database systematically predicted Chinese medicinal herbs with potential to modulate ferroptosis in PCOS. The study identified 22 herbal formulations and 17 of their active components that may exert therapeutic effects by regulating ferroptosis-related gene expression. In this predictive model, *Salvia miltiorrhiza* and *Panax notoginseng* showed high relevance, indicating their possible significance in ferroptosis regulation. At the bioactive ingredient level, salicin B, puerarin, and astragaloside A demonstrated the strongest associations with ferroptosis-related targets (103). These findings offer novel research directions and a theoretical foundation for exploring how TCM may treat PCOS via ferroptosis modulation.

### 5.2 TCM compound

Derived from Ye Tianshi’s Secret Formulas for Gynecological Syndromes, Wenshen Tiaojing Decoction was shown to significantly decrease oxidative stress markers and downregulate ferroptosis-related genes in a PCOS mouse model (104). These findings suggest that its therapeutic effects in PCOS may be mediated through the regulation of oxidative stress and intervention in ferroptosis.

Huanglian Jiedutang is a classical formulation used to clear heat and purge fire. Studies indicate that it exerts multi-target effects on glucose and lipid metabolism by modulating blood glucose and lipid levels, optimizing insulin secretion, improving cortisol profiles, and restoring vascular endothelial function. Key active constituents–such as berberine, baicalin, and gardenin–alleviate ferroptosis-related pathological damage by remodeling gut microbiota composition, improving microecological homeostasis, and modulating ferroptosis-associated signaling pathways (105).

The Bushen Huatan Formula is a classic TCM prescription indicated for kidney deficiency with phlegm-dampness syndrome. It acts by tonifying kidney essence and resolving phlegm-dampness to address related symptoms. Research shows that this formula markedly ameliorates gut microbiota dysbiosis. Through intestinal flora regulation, it elevates short-chain fatty acid levels, activates the intestinal PPARγ pathway, and enhances gut barrier function, thereby effectively mitigating PCOS (106).

Shoutai Pill is a classic TCM formula used for miscarriage prevention, with functions of fortifying the spleen, replenishing the kidneys, and stabilizing pregnancy. In a clinical study involving patients with early threatened miscarriage, treatment with a modified Shoutai Pill significantly upregulated mRNA expression of GPX4 and SLC7A11 and downregulated transferrin receptor 1 expression. These results suggest that the modified Shoutai Pill reduces lipid peroxidation and decreases iron ion accumulation, thereby inhibiting ferroptosis (107).

## 6 Conclusion and perspectives

PCOS, characterized by its complex etiology and heterogeneous clinical manifestations, substantially affects reproductive health and quality of life. Ferroptosis, an emerging form of regulated cell death, has garnered increasing attention in PCOS research. This review synthesizes evidence supporting the role of gut microbiota dysbiosis might be an upstream trigger of ovarian ferroptosis in PCOS. We propose that dysbiosis promotes ferroptosis in ovarian granulosa cells through disrupted iron homeostasis, diminished production of protective microbial metabolites, and systemic inflammation, collectively contributing to the reproductive and metabolic disturbances in PCOS. TCM, including both single herbs and compound formulations, shows promise in modulating gut microbiota composition and function, regulating microbial metabolite production, limiting intestinal iron absorption, and targeting key ferroptosis pathways. These mechanisms may collectively alleviate PCOS symptoms and mitigate long-term risks of metabolic and cardiovascular complications.

Compared with conventional PCOS treatments such as metformin and oral contraceptives, TCM demonstrates unique advantages. Conventional drugs like metformin primarily exert their effects by improving IR, and studies have shown that they can modulate the gut microbiota, albeit with no significant impact on the levels of gut microbial metabolites (108). Oral contraceptives, on the other hand, function mainly through hormonal regulation and have a limited effect on the intestinal microecology; systematic reviews indicate that short-term oral contraceptive use does not significantly alter the gut microbiota in PCOS patients (109). Notably, a study involving women with PCOS undergoing *in vitro* fertilization demonstrated that berberine monotherapy was significantly superior to metformin monotherapy in improving live birth rate, reducing body mass index, decreasing follicle-stimulating hormone consumption, and enhancing gastrointestinal tolerability (110). This finding provides high-level clinical evidence supporting the use of traditional Chinese medicine–either alone or in combination with Western medicine–for PCOS treatment, further highlighting its therapeutic potential and unique value in the comprehensive management of this condition.

In the intervention of ferroptosis-related diseases, current therapeutic strategies face considerable challenges. Although iron chelators can inhibit ferroptosis by reducing the labile iron pool, their long-term use is associated with adverse effects such as ototoxicity, neurotoxicity, and allergic reactions (111). Specific ferroptosis inhibitors like Ferrostatin-1 can effectively block lipid peroxidation but suffer from poor metabolic stability *in vivo*, low bioavailability, and potential off-target effects (112).

In contrast, TCM, characterized by its multi-component, multi-target actions and low toxicity, along with its personalized strategies based on syndrome differentiation and a favorable safety profile, can simultaneously regulate gut microbiota composition, promote the production of beneficial metabolites, inhibit inflammatory pathways, and modulate key ferroptosis molecules. This endows TCM with unique potential in intervening in PCOS through the regulation of ferroptosis by the gut microbiota. However, current TCM research in PCOS primarily focuses on the general modulation of the gut microbiota, with limited attention given to ferroptosis-specific mechanisms. Most studies on ferroptosis in PCOS remain confined to exploring signaling pathways, leaving the gut microbiota–ferroptosis axis insufficiently investigated.

Future studies should prioritize direct functional validation in PCOS models, such as fecal microbiota transplantation to establish causality between specific microbial shifts and ovarian ferroptosis. Integrated use of single-cell sequencing and metabolomics can help delineate precise molecular pathways through which microbial metabolites influence ferroptosis in ovarian cells. While current TCM research remains largely phenomenological, studies on its direct anti-ferroptotic effects and microbiota-mediated mechanisms require substantial expansion. Well-designed preclinical and clinical trials are urgently needed. Future efforts should also focus on identifying active TCM constituents and their molecular targets, systematically mapping their functional networks. These investigations will not only offer novel targets and strategies for PCOS prevention and treatment but also provide a scientific foundation for TCM modernization and the development of microbial-based therapeutics.
