# Correction: The role of gut microbiota mediated ferroptosis in PCOS and the therapeutic potential of Chinese herbal medicine

**DOI:** 10.3389/fmed.2026.1881566

**Published:** 2026-06-05

**Authors:** Yingchun Lv, Dongqi Li, Ning Ding, Hongying Kuang

**Affiliations:** 1 Graduate School, Heilongjiang University of Chinese Medicine, Harbin, Heilongjiang, China; 2 Second Department of Gynecology, The First Affiliated Hospital of Heilongjiang University of Chinese Medicine, Harbin, Heilongjiang, China

**Keywords:** ferroptosis, gut microbiota, polycystic ovary syndrome, traditional Chinese medicine compound formulas, traditional Chinese medicine monomers

The funder Longjiang Science and Technology Talents Chunyan Support Program (Science and Technology Innovation Leading Talent Team)—Innovative Team for Traditional Chinese Medicine Prevention and Treatment of Polycystic Ovary Syndrome in Cold Regions, grant number No. 2022CYCX0026 to (Yingchun Lv, Dongqi Li, Ning Ding, Hongying Kuang) was erroneously omitted.

The original version of this article has been updated.

